# Production of Cement mortars from glass powder and municipal incinerated bottom ash

**DOI:** 10.1038/s41598-024-52298-8

**Published:** 2024-01-18

**Authors:** Park Kumpueng, Lalitsuda Phutthimethakul, Nuta Supakata

**Affiliations:** 1 International Program in Hazardous Substance and Environmental Management, Chulalongkorn University, Bangkok, 10330 Thailand; 2 Department of Environmental Science, Chulalongkorn University, Bangkok, 10330 Thailand; 3 Research Unit (RU) of Waste Utilization and Ecological Risk Assessment, Chulalongkorn University, Bangkok, 10330 Thailand

**Keywords:** Environmental sciences, Materials science

## Abstract

The objective of this research is to find the optimal ratio of glass power (GP) and municipal incinerated bottom ash (MIBA) for producing environmentally friendly interlocking paving blocks. To achieve this, 15 different ratios of mortar samples, sized 5 × 5 × 5 cm, were produced using a 1:3 cement-to-aggregate ratio and a 0.5 water-to-cement ratio. GP was used to substitute cement at 0, 10, and 20% by weight, while MIBA was used to substitute aggregate at 0, 10, 20, 30, and 40% by volume. The samples were divided into two groups and cured with water for 28 and 90 days. Physical testing was performed on the mortar samples after curing. The results show that at 28 days of curing, BA10 and BA20 had compressive strengths of 42.28 and 40.92 MPa respectively, which is higher than the standard for interlocking concrete block (40 MPa) according to TIS 827-2531. At 90 days of curing, GP10BA10, BA10, GP10, GP10BA20, GP20, BA20, and BA30 had compressive strengths of 47.62, 43.63, 43.51, 43.48, 42.73, 42.40, and 40.40 MPa respectively, which also meets the TIS standards.

## Introduction

Si Chang Island, located offshore from Sriracha shore 12 km, is a well-known tourist destination in Chonburi. In 2017, the island welcomed 486,993 visitors. However, as a popular tourist spot, it faces solid waste management issues. The solid waste generation rate on the island is typically around 12–15 tons per day. The local authorities are currently able to collect 17.58 cubic meters of waste per day, but their elimination capacity is only 13.76 cubic meters per day^1^. To manage the waste, the Si Chang municipality uses waste incineration, although this method's effectiveness depends on the components of the municipal solid waste. Staff interviews reveal that during rainfall, the waste collection site cannot feed all the waste to the incinerators due to the high moisture content, leading to solid waste accumulation in the area. Furthermore, glass waste cannot undergo incineration and is either disposed of directly in landfills or left over near the operating site. After the incineration process, the municipal incinerated bottom ash (MIBA) and glass waste are dumped on the island^2^. To mitigate the waste problem on Si Chang Island, it could be possible to study the potential use of MIBA and glass waste to produce construction materials.

Municipal incinerated bottom ash (MIBA) is a byproduct of waste incineration that is currently being disposed of by dumping around two tons per month on the island^2^. However, instead of this wasteful disposal method, MIBA can be utilized as a valuable material. One promising approach, which has been thoroughly researched, is the use of MIBA as aggregate in the construction industry^3,4^. This study aims to explore the use of MIBA as an aggregate in the production of interlocking paving blocks. By partially substituting natural aggregate with MIBA, this sustainable solution can help mitigate waste management issues on Si Chang Island and reduce the depletion of valuable natural resources.

Glass waste, left over after the sorting process prior to solid waste incineration, is a type of municipal solid waste that cannot be incinerated. On Si Chang Island, the glass waste, consisting of beer bottles, vitamin bottles, and food ingredient bottles, is sorted and kept within the waste station area before being sent to the dumpsite^2^. Researchers have found that glass waste, with its primary chemical composition of silica, can be ground into a fine powder and utilized as coarse aggregate, fine aggregate, and binder^5–7^. In this study, the glass waste will be ground into a fine powder and used as a partial substitute for Portland cement, serving as a pozzolan, to increase the reactivity of the glass. Glass, which is produced by melting silica, soda ash, and calcium carbonate at high temperatures and solidifying without crystallization, can be produced in various shapes and sizes and recycled multiple times without significant alteration of physical or chemical properties. The amorphous structure and high vitreous silica content of glass suggest its potential use as a supplementary cementitious material.

Supplementary cementitious materials also known as pozzolanic materials are inorganic materials that become solid when mixing with calcium hydroxide from hydrated lime, or material that can release calcium hydroxide, like Portland cement. Pozzolanic material can be either natural or artificial, which normally classified by the preparing method. Natural pozzolanic material requires only grinding with no further treatment, modification, but artificial pozzolanic materials require pre-treatment to modify chemical or physical properties because they have weak or no pozzolanic properties^8^. In this study, glass waste was crushed to be glass powder (GP) which is considered as supplementary cementitious material.

The properties of the pozzolanic materials, consisting of vitreous silica content and fineness, will determine the rate of reaction. Moreover, the amount of lime released during the Portland cement hydration reaction will affect the pozzolanic materials. The chemical composition comparison of a variety of supplementary cementitious material with the Portland cement has been shown in the ternary diagram of Aïtcin^9^.

This study introduces a new waste management approach by exploring the combined use of glass powder (GP) and municipal incinerated bottom ash (MIBA) in interlocking paving blocks with high level of substitution levels, and also testing synergistic effects of these waste materials. The results suggest promising opportunities to decrease landfill waste, reduce carbon emissions, and improve the sustainability of paving block production, especially in island communities with waste management issues. This work opens avenues for wider acceptance of waste-derived materials in construction, potentially inspiring innovative solutions for a more sustainable future.

## Results

### General appearance of raw materials

**Figure 1:**
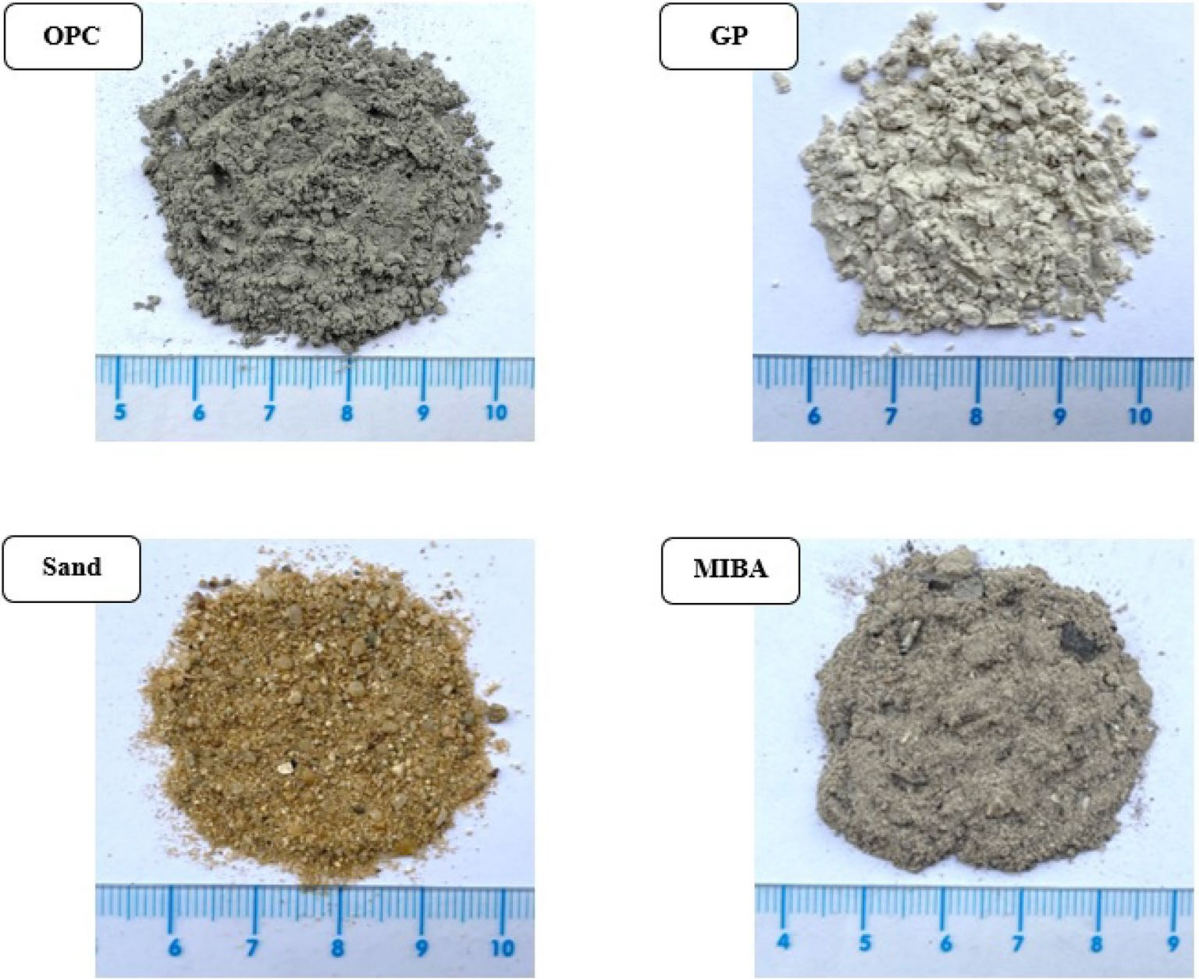
Appearance of raw materials.

The visual characteristics of the raw materials were depicted in Fig. 1. The figure illustrated that the ordinary Portland cement (OPC) had a dark grey powdery appearance, whereas GP appeared as a white powdery substance with comparable fineness to OPC. In terms of the fine aggregates used in this study, sand and MIBA had distinct appearances. Sand exhibited coarser particles that were easily visible, while MIBA had a finer texture.

### Particle size distribution of binder

**Figure 2:**
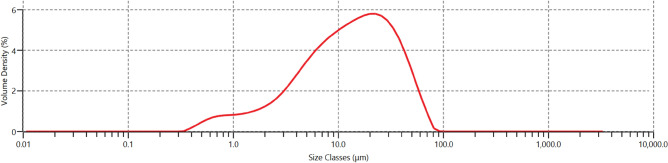
Particle size distribution of cement.

**Figure 3:**
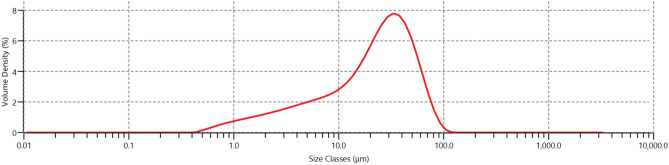
Particle size distribution of glass powder.

Figures 2 and 3 present the results of the particle size distribution analysis of cement and glass powder, respectively. The findings indicate that cement particles are finer than glass powder particles, with a D_50_ value of 13.1 μm. In contrast, glass powder particles have a D_50_ value of 22.8 μm.

### Particle size distribution of fine aggregate

**Figure 4:**
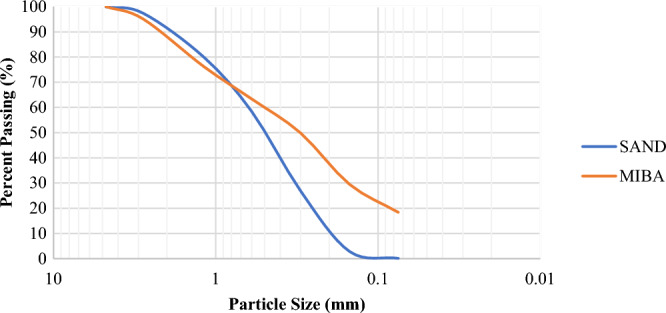
Particle size distribution of sand and MIBA.

The sieve analysis method was used in this research to evaluate the particle size distribution of sand and MIBA, and the results were presented in Fig. 4. It was observed that MIBA has a smaller particle size than sand, as the D_50_ value for MIBA.

### Chemical composition of raw material

Table 1 presents the main chemical components of OPC.

**Table 1:** Chemical composition of ordinary Portland cement (OPC), glass powder (GP), sand, and municipal incinerated bottom ash (MIBA).

Oxides	Content (wt%)			
	OPC	GP	Sand	MIBA
SiO_2_	16.6	67.2	73.6	17
Al_2_O_3_	3.42	2.4	4.72	4.22
CaO	62.7	10.2	868 (PPM)	31.3
Fe_2_O_3_	3.22	0.633	0.765	2.07
Na_2_O	0.223	13.6	0.266	3.57
MgO	1.14	2.68	868 (PPM)	2.31
TiO_2_	0.227	0.113	0.125	1.19
SO_3_	3.57	725 (PPM)	105 (PPM)	3.04
P_2_O_5_	490 (PPM)	287 (PPM)	345 (PPM)	1.96
MnO	557 (PPM)	238 (PPM)	87.4 (PPM)	875 (PPM)
ZrO_2_	63.7 (PPM)	235 (PPM)	141 (PPM)	156 (PPM)
BaO	N/D	231 (PPM)	359 (PPM)	729 (PPM)
Cr_2_O_3_	N/D	229 (PPM)	N/D	204 (PPM)
Cl	252 (PPM)	197 (PPM)	N/D	5.22
ZnO	353 (PPM)	171 (PPM)	N/D	0.22
SrO	400 (PPM)	134 (PPM)	34.7 (PPM)	402 (PPM)
CuO	237 (PPM)	97.2 (PPM)	N/D	0.118
NiO	N/D	39.1 (PPM)	N/D	81.6 (PPM)
PbO	N/D	33.5 (PPM)	27.4 (PPM)	300 (PPM)

### The mineralogic phase of raw materials

**Figure 5:**
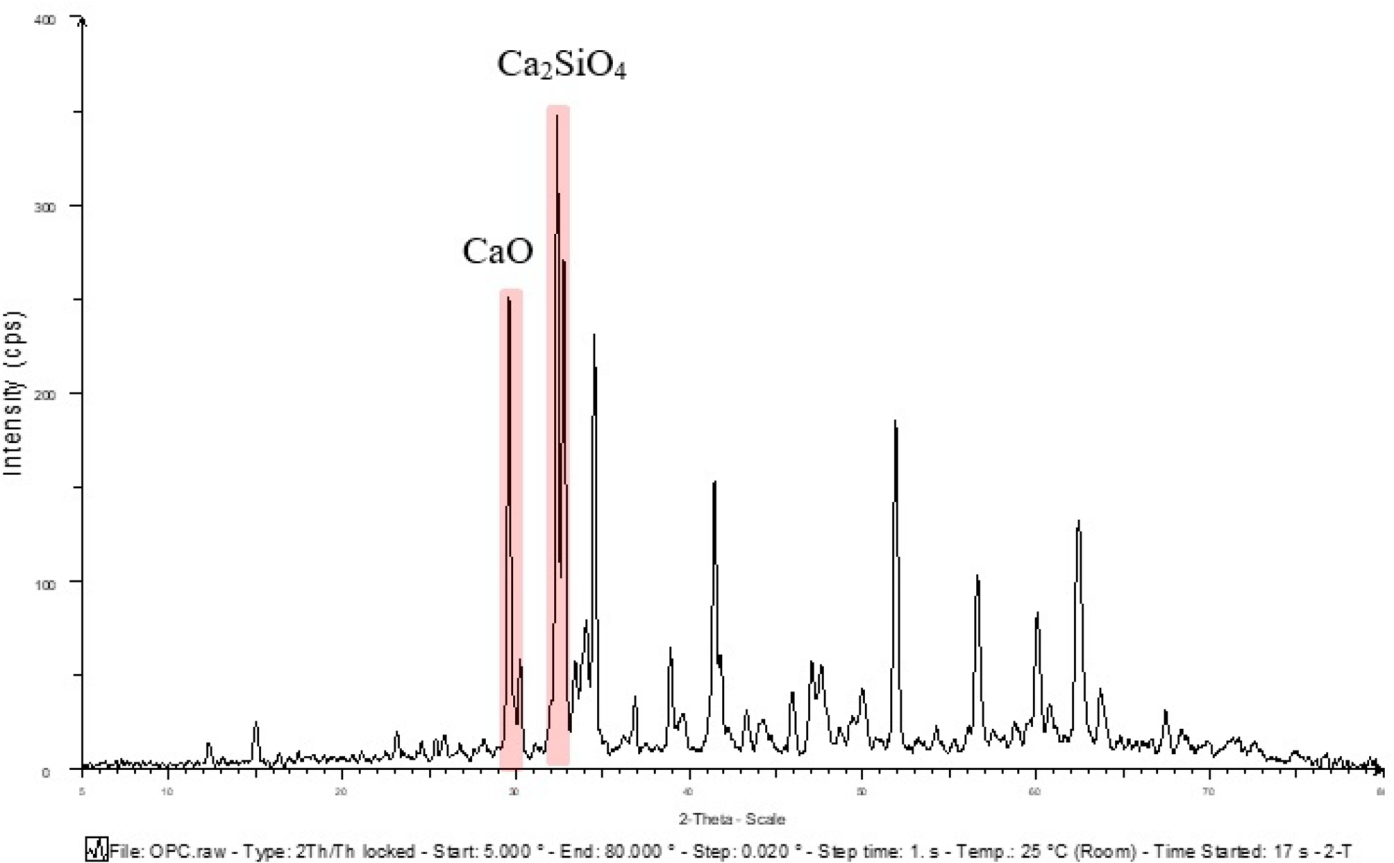
Mineralogical phases of Portland cement.

**Figure 6:**
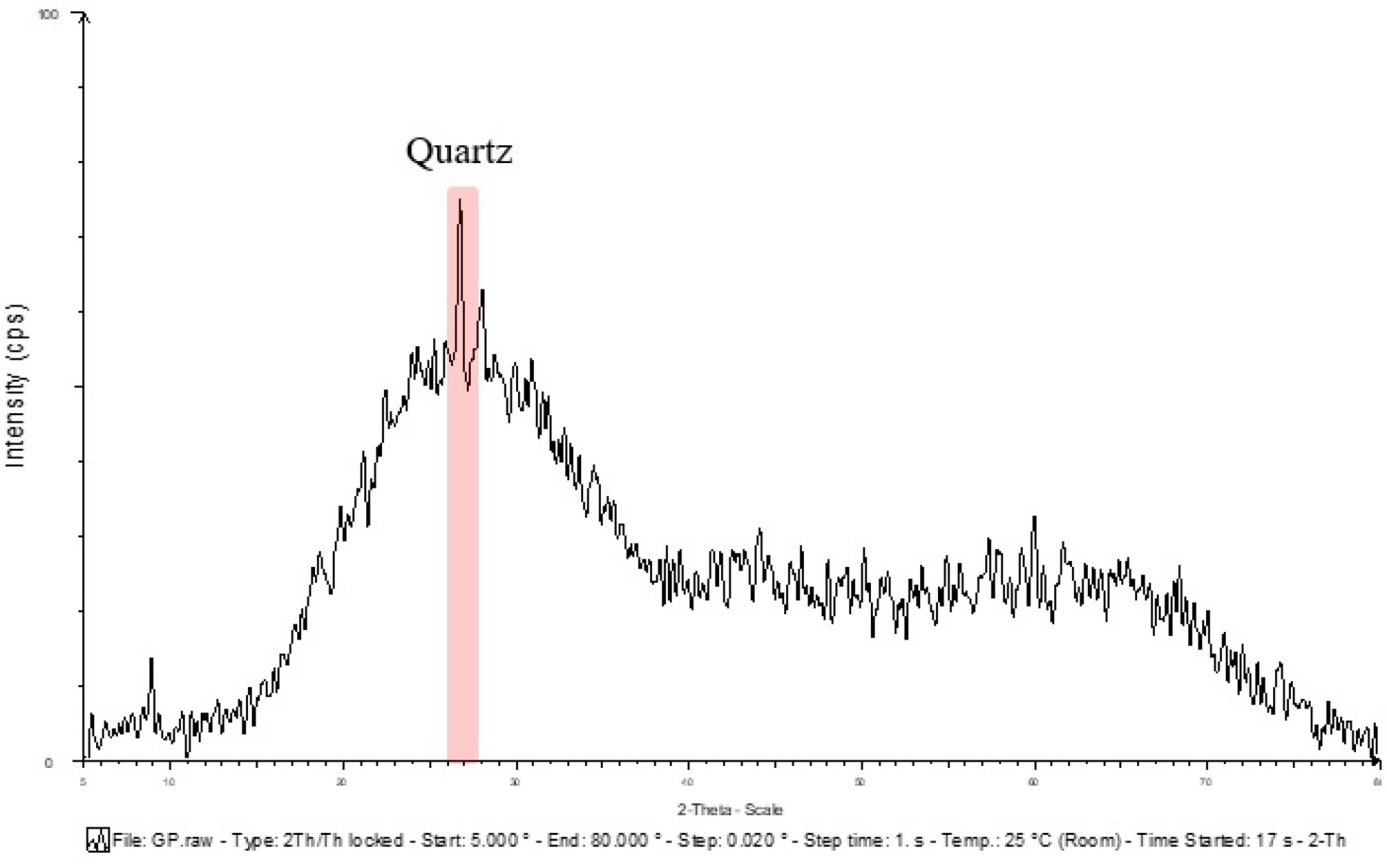
Mineralogical phases of glass powder.

**Figure 7:**
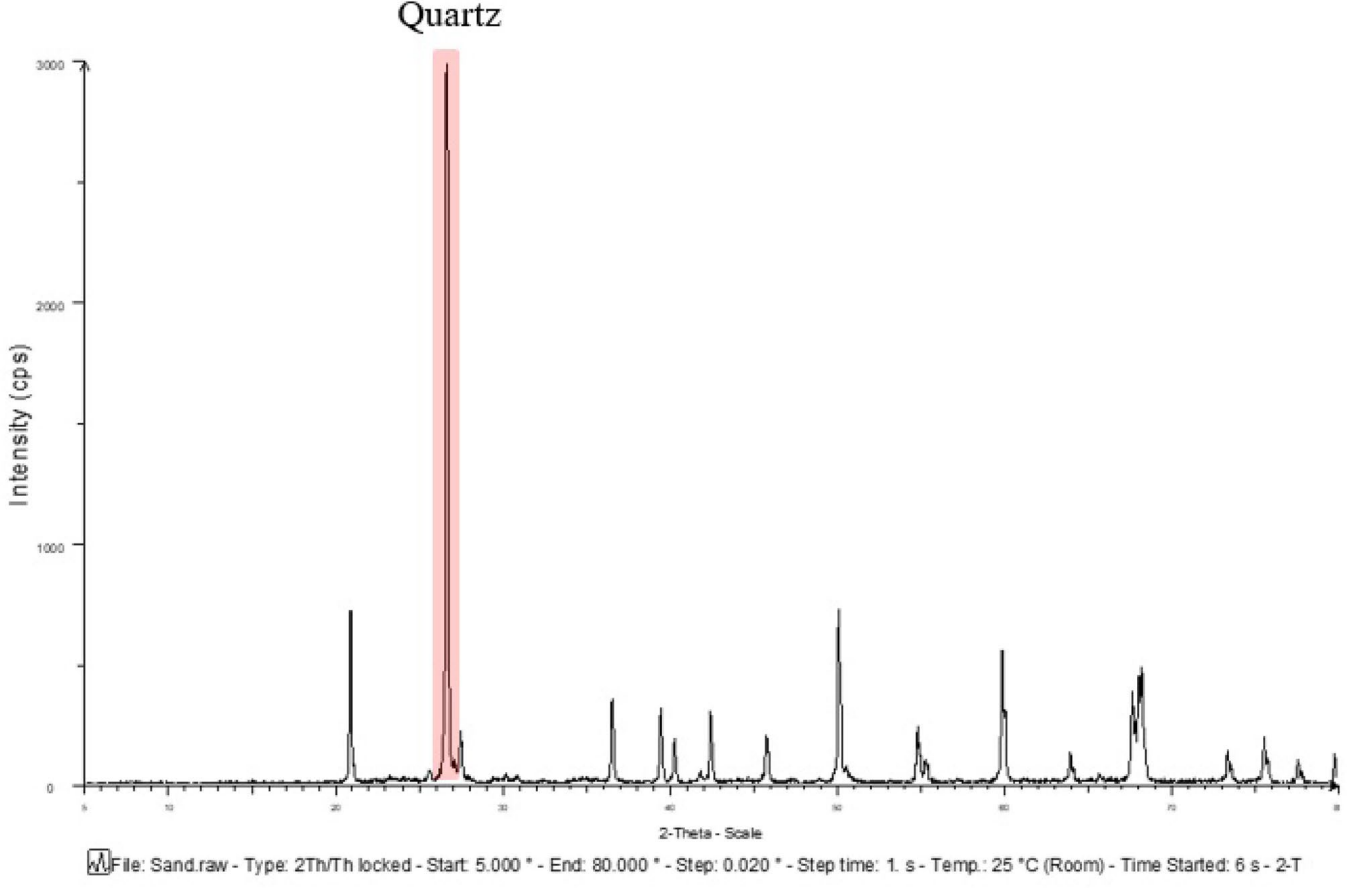
Mineralogical phases of sand.

**Figure 8:**
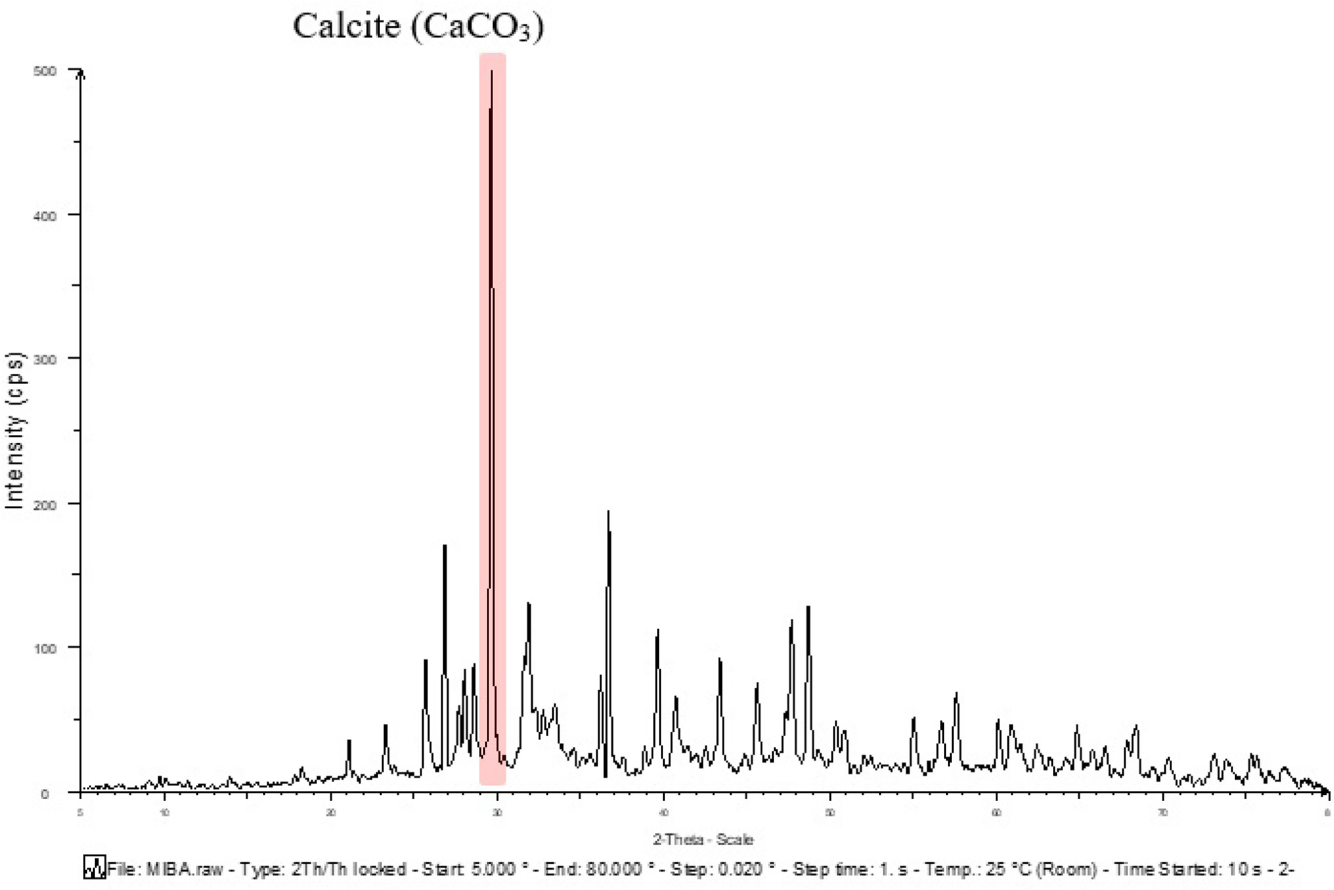
Mineralogical phases of municipal incinerated bottom ash.

**Figure 9:**
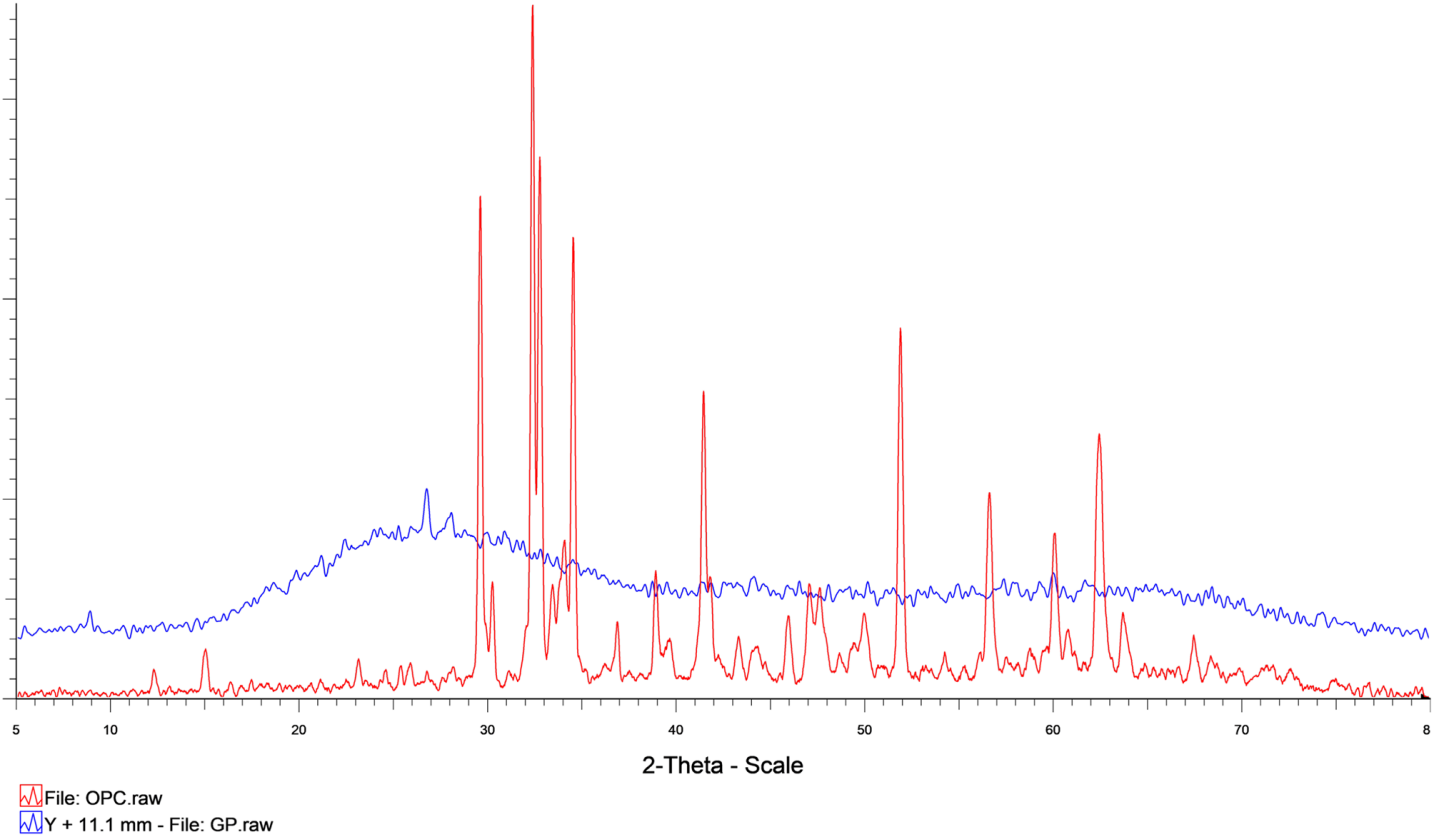
Comparative mineralogical phases graph of Portland cement and Glass powder.

The X-ray diffraction (XRD) method was used to analyze the mineralogical phase of the raw materials, and the results for each material were presented in Figs. 5, 6, 7, and 8. A comparison of the mineralogical phase between OPC and GP was shown in Fig. 9, with calcium silicate oxide (Ca_2_SiO_4_) being the major phase in OPC and amorphous silica oxide (SiO_2_) being the major phase in GP.

**Figure 10:**
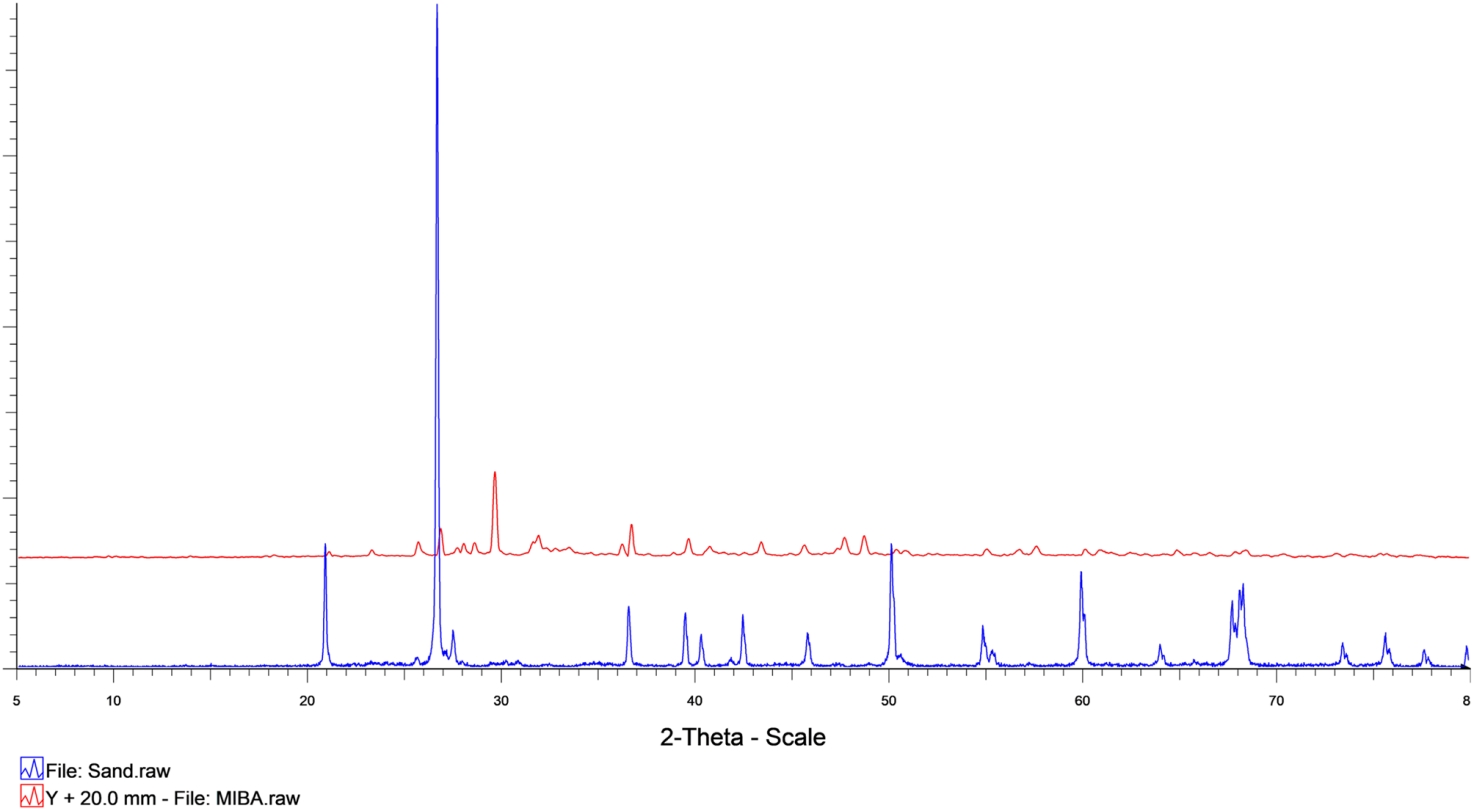
Comparative mineralogical phases graph of sand and municipal incinerated bottom ash.

The mineralogical phase of sand and MIBA was also analyzed and compared, and the results were shown in Fig. 10. The major mineralogical phase in sand was quartz or crystalline silica oxide (SiO_2_), while MIBA had calcite oxide (CaCO_3_) as the major phase. The XRD results show that the phases and species of minerals are related to the chemical compositions obtained from the XRF analysis.

### The microstructure characterization and element

**Figure 11:**
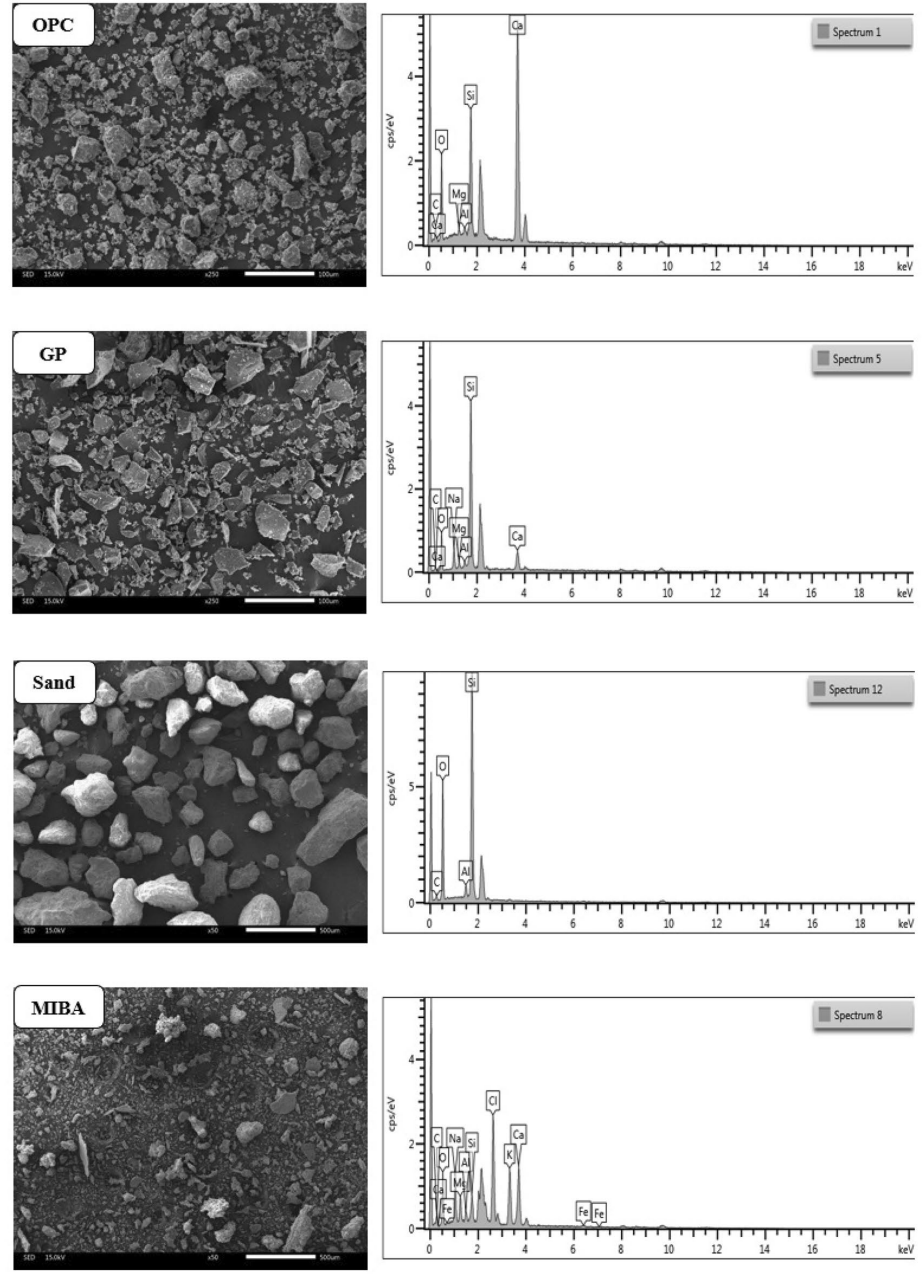
Micro-structure characterization of raw materials.

The scanning electron microscope (SEM) method using Jeol JSM-IT300 was utilized to examine the microstructure of all raw materials, including OPC, GP, sand, and MIBA. The element composition of the materials was also analyzed through the use of an Energy Dispersive X-ray Spectrometer (Oxford X-Max 20), as shown in Fig. 11 alongside the SEM images. The SEM images revealed that OPC and GP had similar particle sizes and shapes. However, when comparing the particle sizes and shapes of sand and MIBA, it was found that MIBA had smaller particles and greater porosity than sand.

### The leaching of heavy metals from MIBA

The leaching of heavy metals from the municipal incinerated bottom ash was assessed using the TCLP method^10^, and the findings are presented in Table 2. The levels of heavy metals were compared against the permissible limits outlined in the US code of federal regulations^11^ and soil quality standards from the Thai pollution control department^12^. The results indicate that the concentration of heavy metals in the municipal incinerated bottom ash falls within the acceptable range. Therefore, the municipal incinerated bottom ash can be considered non-hazardous waste and be utilized.

**Table 2:** The leaching of heavy metals from MIBA.

Heavy metal	MIBA (mg/L)	Regulatory level (mg/L)*	Soil quality standards (mg/kg)**
Ba	0.638 ± 0.169	100.0	–
As	Not detected	5.0	3.9
Co	Not detected	–	–
Cd	0.003 ± 0.000	1.0	37
Fe	Not detected	–	–
Cr	0.228 ± 0.028	5.0	300
Mn	Not detected	–	1800
Cu	0.188 ± 0.058	–	–
Se	0.010 ± 0.000	1.0	390
Zn	0.013 ± 0.008	–	–
Ni	Not detected	–	1600
Pb	0.011 ± 0.003	5.0	400

*Identification and Listing of Hazardous Waste^11^.

**Pollution Control Department^12^.

### Properties of mortar sample

#### Characterization of mortar sample

##### General appearance of mortar samples

**Figure 12:**
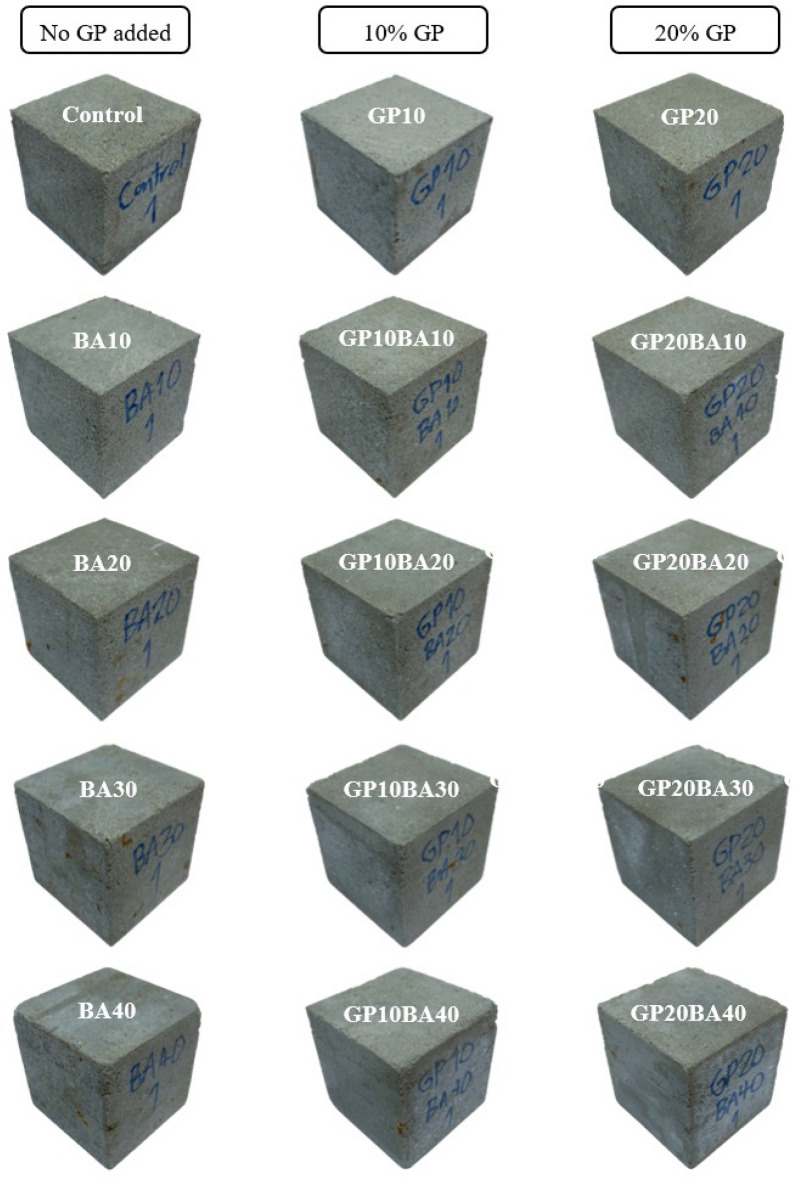
General appearance of 28 days-cured mortar samples.

**Figure 13:**
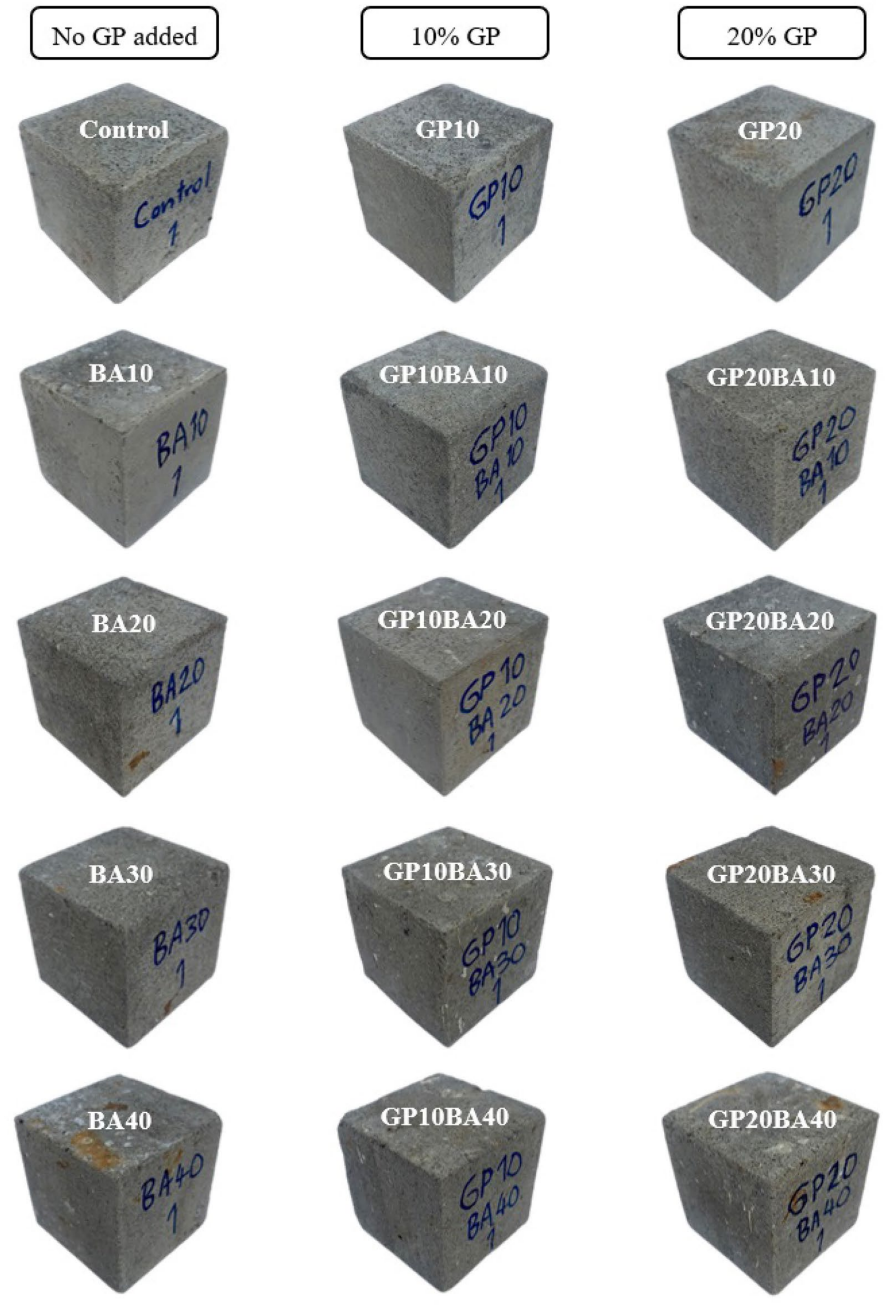
General appearance of 90 days-cured mortar samples.

Figures 12 and 13 display the visual appearance of the mortar samples after being cured for 28 and 90 days. The mortar samples were divided into three categories based on the proportion of glass powder substitution: no substitution, 10% substitution, and 20% substitution.

The external appearance and surface of the samples were similar between the two curing durations for each proportion. However, the color of the samples varied considerably across the proportions. As the proportion of MIBA used in substitution of sand increased, the surface of the samples tended to become darker gray.

##### Compressive strength mortar samples

**Figure 14:**
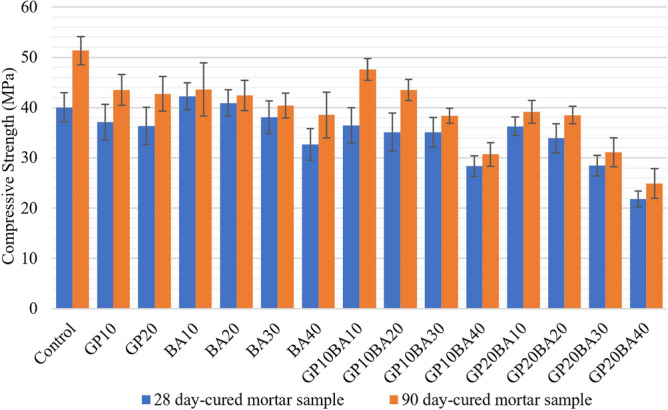
Comparative compressive strength graph of mortar samples cured for 28 and 90 days.

The mortar samples were categorized based on the curing time into two groups: those cured for 28 days and those cured for 90 days. The results are presented in Fig. 14.

For the 28-day cured mortar samples, two ratios met the standard requirements for interlocking paving block^13^, which is TIS827-2531 (> 40 MPa). These two ratios are BA10 and BA20, which had compressive strengths of 42.28 and 40.92 MPa, respectively. Meanwhile, the compressive strength of the 90-day cured mortar samples was better than the 28-day cured ones. Seven ratios of mortar samples cured for 90 days met the requirements of TIS827-2531 (> 40 MPa). The highest compressive strength was achieved by GP10BA10, with 47.62 MPa, followed by BA10 (43.63 MPa), GP10 (43.51 MPa), GP10BA20 (43.48 MPa), GP20 (42.73 MPa), BA20 (42.40 MPa), and BA30 (40.40 MPa), respectively.

The impact of GP and MIBA on compressive strength in mortar samples after 28 days was analyzed using a two-way ANOVA. It demonstrated that no significant interaction was found between the effects of GP and MIBA on compressive strength, with F (8, 60) = 2.03 and *p* = 0.06. This implies that using a combination of glass powder and municipal incinerated bottom ash does not have any effect on the compressive strength of the samples. However, the main variables, GP and MIBA, have a *p* value of 0.000, indicating that different quantities of glass powder and municipal incinerated bottom ash have a significant impact on the compressive strength of the sample at a 0.01 level.

The data analysis of the compressive strength after 90 days indicates that the results differ from those obtained after 28 days. According to the simple main effects analysis, both GP and MIBA were significantly different, with a *p* value of 0.00. Additionally, an interaction between GP and MIBA was observed, with F(8,60) = 4.87 and *p* = 0.00, indicating that the curing time had an impact on the compressive strength and that the combination of GP and MIBA affects the compressive strength significantly. To identify the significant ratios, a one-way ANOVA test is required to analyze the simple effects between samples.

The Tukey test was used to conduct multiple comparisons of the compressive strength at 90 days between samples via one-way ANOVA analysis. The results revealed that the control samples, which did not use any glass powder or municipal incinerated bottom ash, were significantly different from all other samples, except for GP10BA10.

##### Water absorption of mortar samples

**Figure 15:**
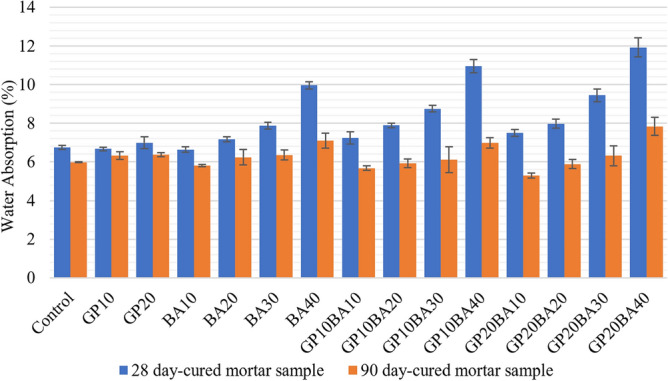
Comparative water absorption graph of 28 and 90 days-cured mortar samples.

The water absorption of mortar samples was examined and compared between those cured for 28 days and 90 days. This analysis is shown in Fig. 15. The results suggest that mortar samples cured for 28 days tend to have a higher water absorption rate than those cured for 90 days. For the 28-day cured samples, the BA 10 sample had the lowest water absorption rate at 6.64%, while the GP20BA20 sample had the highest water absorption rate at 11.92%. Substituting the binder and aggregate can increase the water absorption rate of mortar samples as the level of aggregate substitution increases. However, the results for the 90-day cured samples show a different trend in water absorption. The GP20BA10 sample had the lowest water absorption rate at 5.29%, while the GP20BA40 sample had the highest water absorption rate at 7.84%.

A simple main effects analysis of water absorption after 28 days revealed that both GP and MIBA had a significant effect (*p* value = 0.02), and there was also a significant difference when using both GP and MIBA, with F(8,60) = 2.62, *p* = 0.16. This indicates that the interaction between GP and MIBA had a significant effect on water absorption. In addition, a one-way ANOVA test was required to analyze the simple effects between samples and determine the significant ratio.

The Tukey test, conducted through one-way ANOVA analysis, was used to analyze the multiple comparisons of water absorption after 28 days between samples. The results indicate that the control sample (using 0% glass powder and 0% municipal incinerated bottom ash) has a different level of significance from only the GP20BA40 sample.

The data analysis results for water absorption at 90 days shows that using only GP did not have a significant effect on water absorption (*p* value = 0.16), but using only MIBA resulted in a significant difference (*p* value = 0.00), similar to using both wastes, with F(8,60) = 16.5 and *p* = 0.00. Further analysis is needed to determine the significant ratios using a one-way ANOVA test to investigate the simple effects between the samples.

To investigate the multiple comparisons of water absorption at 90 days between samples, the Tukey test was conducted using one-way ANOVA analysis. It is noteworthy that most of the samples did not significantly differ from the Control sample (using 0% glass powder and 0% municipal incinerated bottom ash), except for GP10BA10, GP20BA10, and GP20BA40.

##### Density of mortar sample

**Figure 16:**
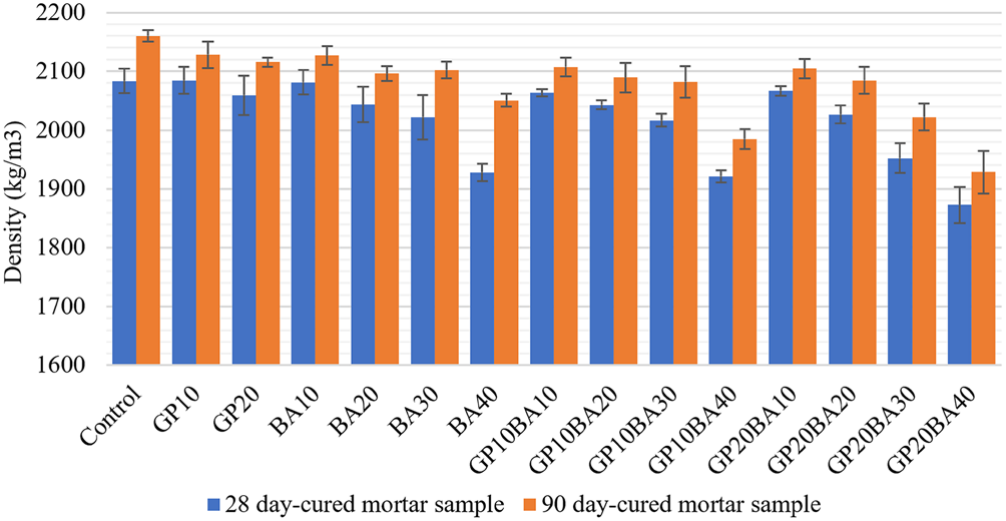
Comparative density graph of 28 and 90 days-cured mortar samples.

The density of mortar samples that were cured for 28 days and 90 days was compared in Fig. 16. It was found that the density tends to decrease as the amount of raw material being replaced by waste in the production of mortar samples for both curing periods increases. The results also indicated a correlation between compressive strength and density. For the mortar samples cured for 28 days, the GP10 sample had the highest density of 2084.71 kg/m^3^, followed by the control sample (2083.33 kg/m^3^) and the BA10 sample (2081.10 kg/m^3^), respectively. For the mortar samples cured for 90 days, the control had the highest density at 2159.73 kg/m^3^, followed by GP10 (2128.06 kg/m^3^) and BA10 (2126.62 kg/m^3^), respectively. Furthermore, the results of both 28 days-cured and 90 days-cured mortar samples indicated that GP20BA40 has the lowest density compared to other samples, with a density of 1872.78 and 1928.72 kg/m^3^, respectively.

The results of the density analysis of samples at 28 days revealing a significant effect on the density of samples when GP and MIBA were used (*p* value = 0.00). Additionally, an interaction between GP and MIBA was observed to have a significant effect, as indicated by F(8,60) = 2.42, *p* = 0.24. To determine the significant ratio, a one-way ANOVA test was necessary to analyze the simple effects between samples.

The Tukey test via one-way ANOVA analysis was performed to analyze the multiple comparisons of density at 28 days for each sample. The results shown that almost half of the samples significantly differed from the Control sample (0% glass powder and 0% municipal incinerated bottom ash), including BA30, BA40, GP10BA30, GP10BA40, GP20BA20, GP20BA30, and GP20BA40.

The density of the samples at 90 days was also examined and yielded results similar to those of the 28-day samples. Both GP and MIBA had a significant effect on the density with a *p* value of 0.000, and an interaction between GP and MIBA was observed, with F(8,60) = 7.11 and *p* = 0.00. Additionally, a one-way ANOVA test was performed to examine the simple effects between samples and to identify any significant differences.

The multiple comparisons of density at 90 days were evaluated using the Tukey test and one-way ANOVA analysis. The results are presented that with the exception of BA10, GP10, and GP20, almost all of the samples did not significantly differ from the control sample (using 0% Glass powder and 0% municipal incinerated bottom ash).

##### The microstructure characterization of mortar samples

**Figure 17:**
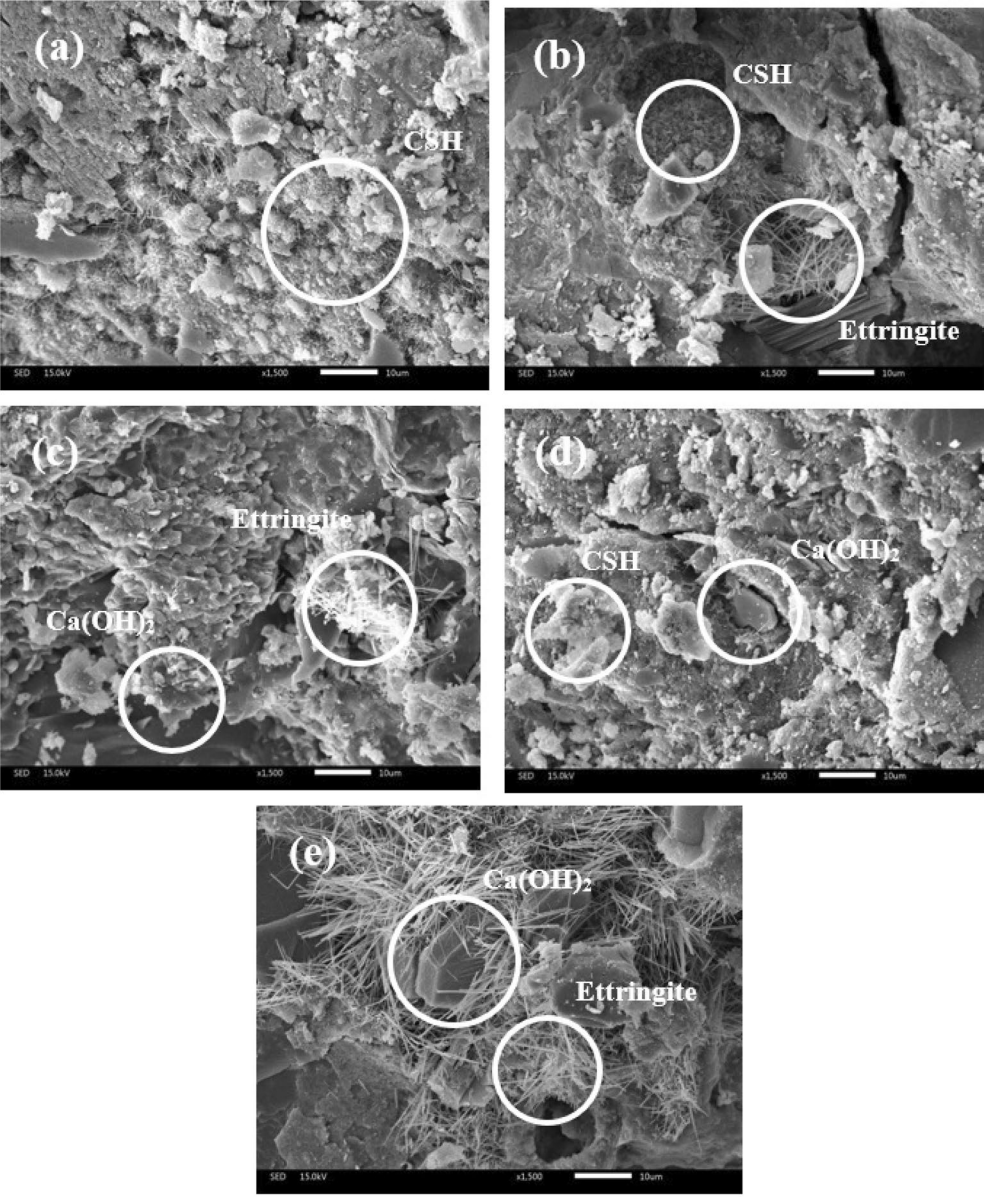
Microstructure of 28 days-cured mortar sample of Control (**a**), GP20 (**b**), BA40 (**c**), BA10 (**d**), and GP20BA40 (**e**).

**Figure 18:**
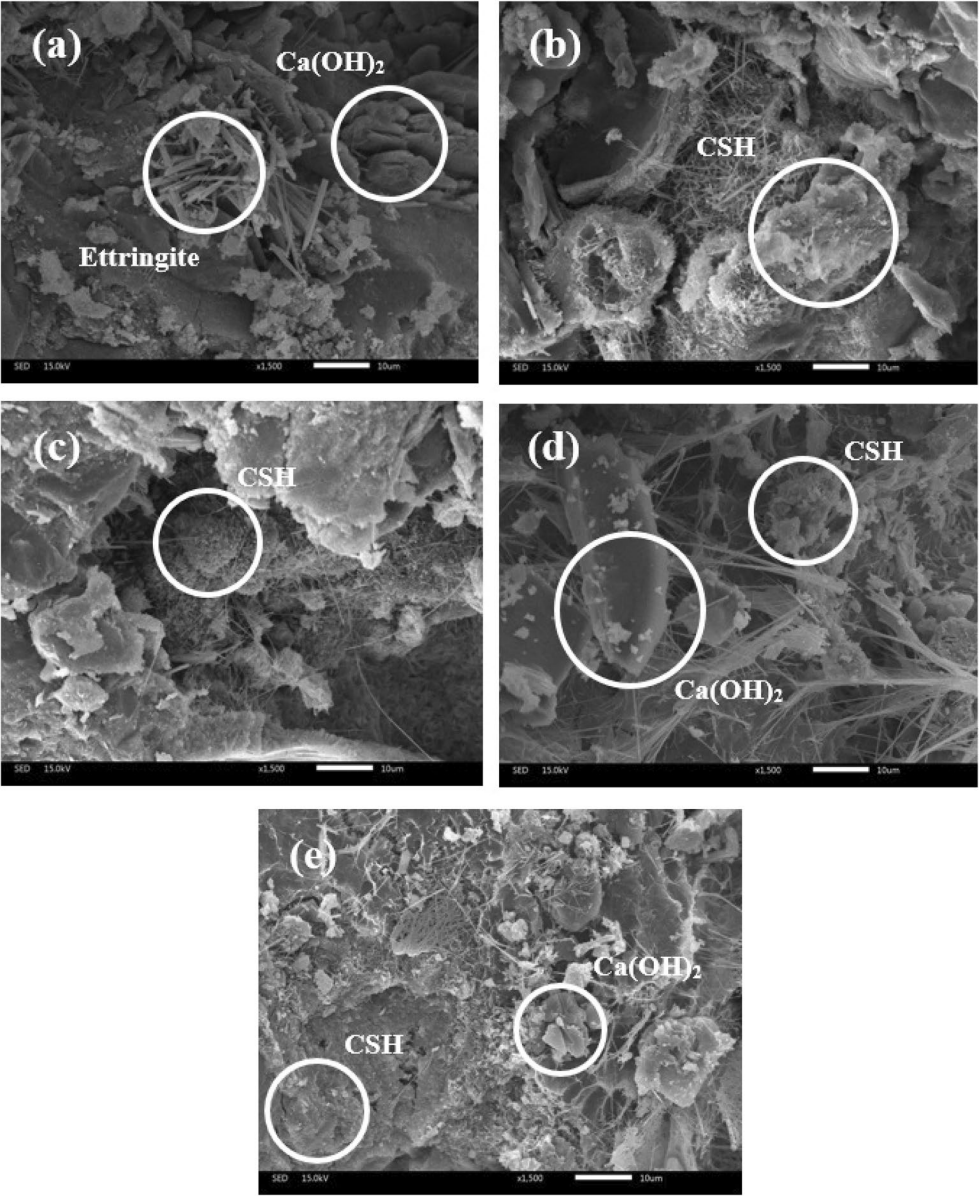
The microstructure of 90 days-cured mortar sample of Control (**a**), GP20 (**b**), BA40 (**c**), GP10BA10 (**d**), and GP20BA40 (**e**).

The Scanning Electron Microscope (JEOL JSM-IT300) was used to investigate the microstructure of the mortar samples at a magnification of 1500x. The analysis focused on two curing periods: 28 days and 90 days. The samples selected for analysis were based on various criteria, including samples without waste, those with the highest substitution by GP, those with the highest substitution by MIBA, best and worst performance samples. For 28 days-cured samples, the selected samples were control, GP20, BA40, BA10, and GP20BA40, as shown in Fig. 17. For 90 days-cured samples, the selected samples were control, GP20, BA40, GP10BA10, and GP20BA40, as shown in Fig. 18. The microstructure analysis revealed the presence of Calcium silicate hydrate (CSH) and Ettringite in all the samples, which are products of the cement hydration reaction. CSH provides bonding between the aggregates and gives strength to the sample. Ettringite has a needle-shaped particle that forms as a result of sulfate ions in gypsum reacting with Tricalcium aluminate (C_3_A) and water^14^. In addition, the analysis showed the presence of hexagonal-shaped calcium hydroxide crystals on the surface of the sample^15^.

##### Mineralogical phases of mortar samples

**Figure 19:**
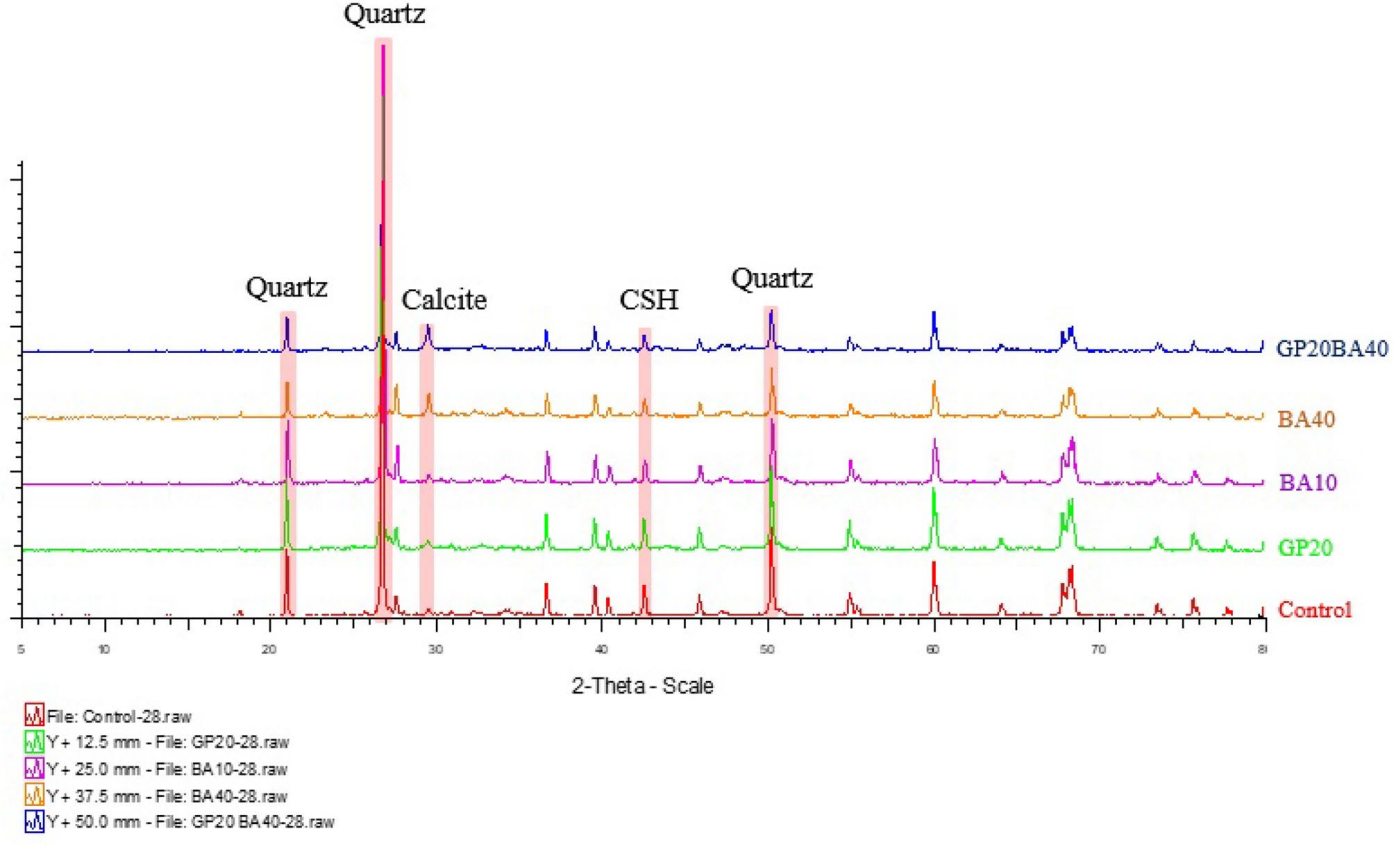
Mineralogical phases of 28 days-cured mortar samples.

**Figure 20:**
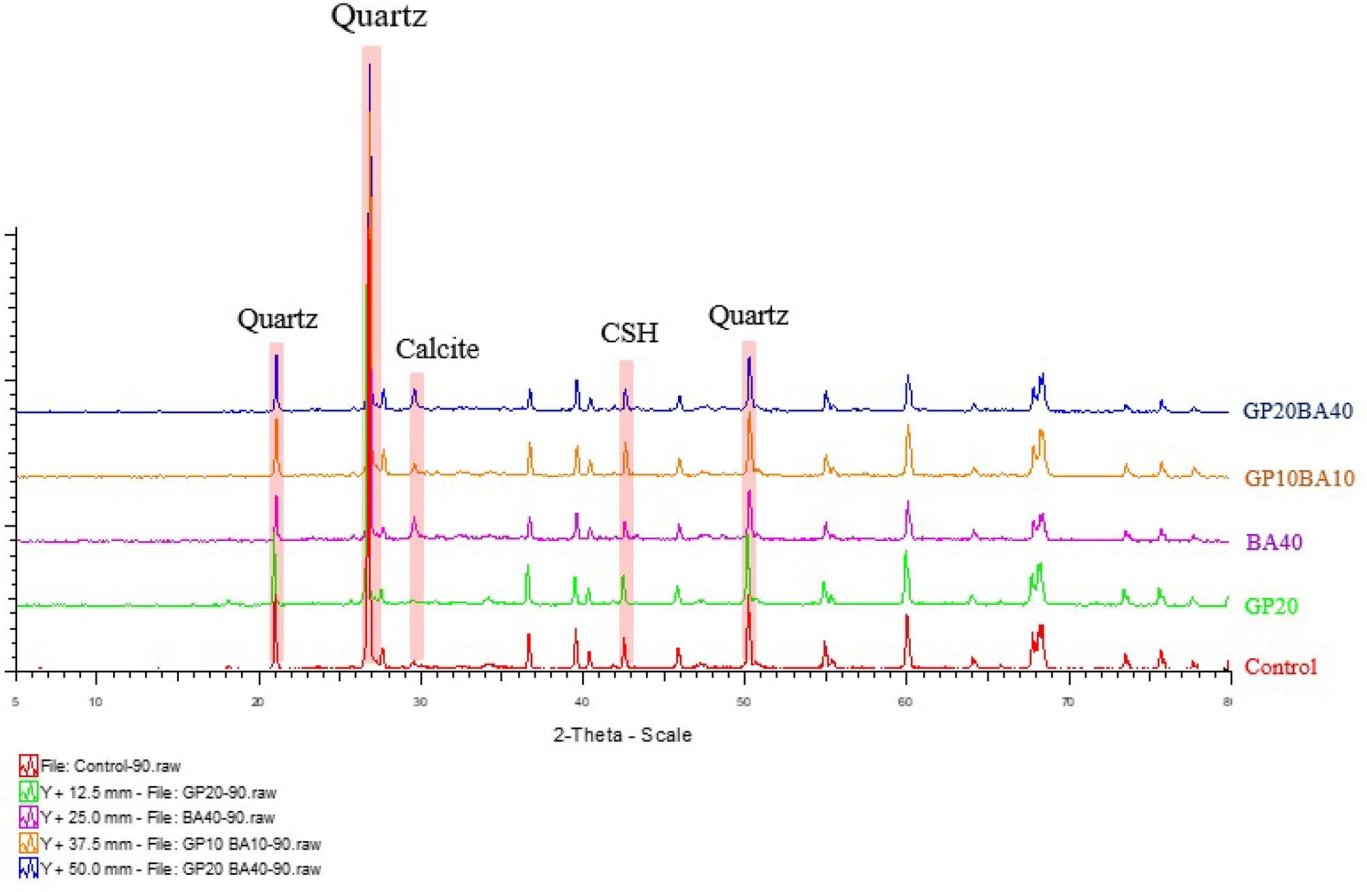
Mineralogical phases of 90 days-cured mortar samples.

The mineral phases of mortar samples cured for 28 days and 90 days were examined and compared, which are presented in Figs. 19 and 20, respectively. The analysis revealed that calcium silicate hydrate (CSH) is the primary mineral phase in the mortar samples after the cement hydration process, which appears at 42.7° (2θ)^16^. In addition, the XRD results demonstrated that the major mineral components of the mortar samples are quartz (SiO_2_) and calcite (CaCO_3_).

## Methods

Ordinary Portland cement (OPC) type 1 was used in this study along with glass powder (GP) which was obtained from a waste recycling facility by crushing glass waste using a ball mill and then sieved through a 45-micron sieve to obtain desired fineness.

Sand and municipal incinerated bottom ash (MIBA) were used as aggregates. MIBA obtained from the incinerator on the Si Chang Island in the Chonburi province of Thailand which was a mixture of fly ash and bottom ash. Sand and MIBA were sieved through a No.4 to eliminate the size exceed than 4.75 mm prior to use.

Cement, GP, sand, and MIBA were tested for physical and chemical properties. Particle size distribution was conducted using a Laser particle size distribution analyzer (MALVERN Mastersizer 3000, United Kingdom). Chemical compositions were analyzed using the X-ray fluorescence spectrometer method (Bruker model S8 Tiger). X-ray diffraction method (XRD) was used to determine the mineralogical phase of material using Bruker AXS model S4 Pioneer, Karlsruhe, Germany. The microstructure was also investigated by using a scanning electron microscope (JEOL JSM-IT300). Moreover, MIBA was analyzed for the hazardous substances using toxicity characteristic leaching procedure (TCLP) for analysis^10^.

### Preparation of mortar samples

**Figure 21:**
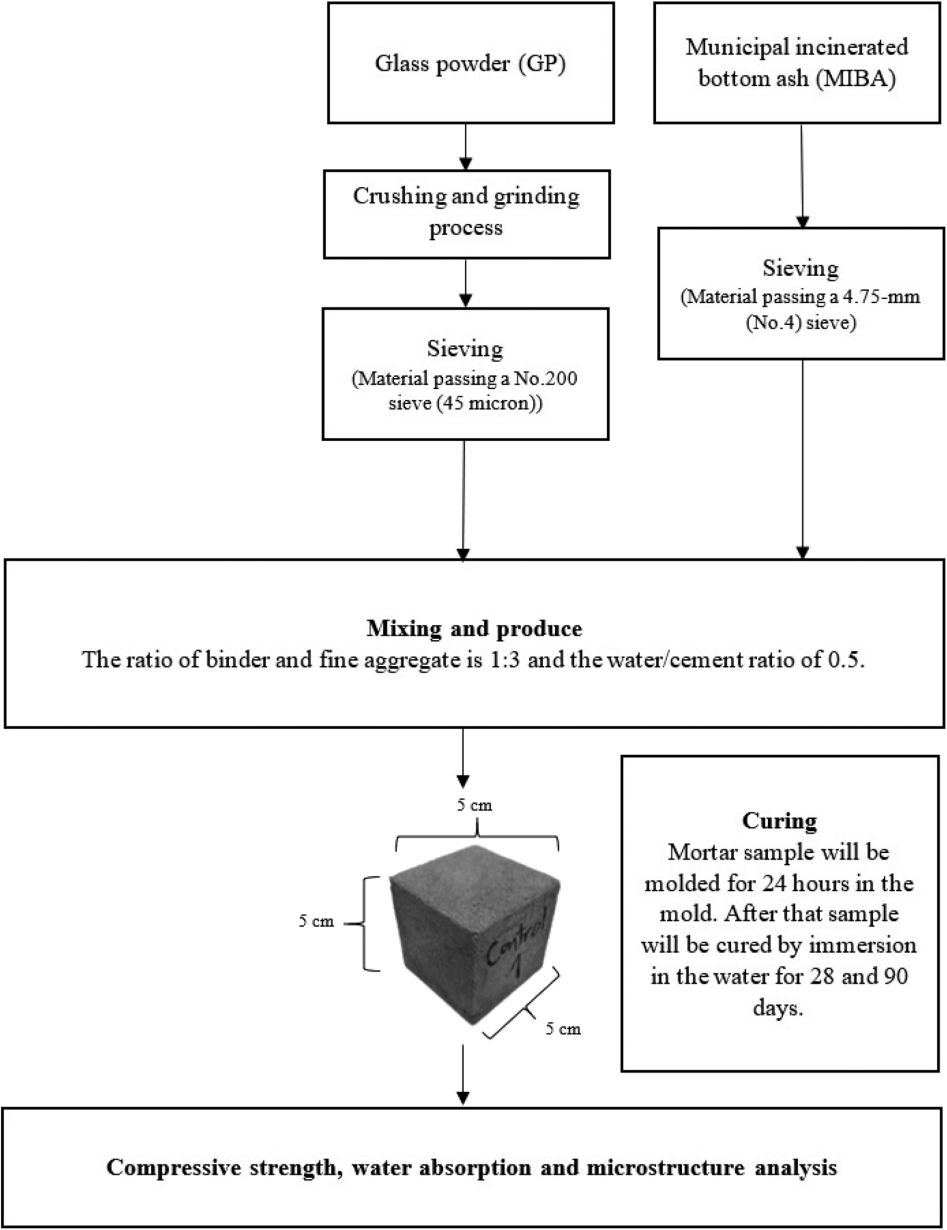
Flowchart of the interlocking paving block production.

The mortar sample was prepared in accordance with ASTM C109 with the proportion of materials for mortar production used as shown in Table 3. The ratio of binder and fine aggregate is 1:3 using water/binder ratio of 0.5. Mortar sample will be molded for 24 h in the mold. After that sample will be cured by immersion in the water for 28 and 90 days. Each formulation has 10 replicates per formula for strength, water absorption, and microstructure analysis. The overall process of mortar production was summarized as shown in Fig. 21. The mortar sample were tested for compressive strength and water absorption according to ASTM C109^17^ and ASTM C642^18^, respectively.

**Table 3:** Mortar formulations.

Treatments	1		3		Water/Cement
	Binder		Fine aggregate		
	Cement (%)	Glass powder (%)	Sand (%)	MIBA (%)	
Control	100	0	100	0	0.5
GP10	90	10	100	0	
GP20	80	20	100	0	
BA10	100	0	90	10	
BA20	100	0	80	20	
BA30	100	0	70	30	
BA40	100	0	60	40	
GP10BA10	90	10	90	10	
GP10BA20	90	10	80	20	
GP10BA30	90	10	70	30	
GP10BA40	90	10	60	40	
GP20BA10	80	20	90	10	
GP20BA20	80	20	80	20	
GP20BA30	80	20	70	30	
GP20BA40	80	20	60	40

## Discussion

The compressive strength of mortar samples was as expected when glass powder was partially substituted for Portland cement. Nahi, Leklou^19^, using glass powder to partially replace Portland cement can enhance the flowability of the mortar by increasing the water-to-cement ratio, as glass powder is non-absorbent. The early-stage compressive strength of 28-day cured mortar samples decreased due to the dilution effect, but the 90-day cured mortar samples showed an increase in compressive strength as the cement continued to undergo hydration reactions. The pozzolanic reaction had a minor contribution to the compressive strength, while the hydration reaction had a major contribution. Moreover, glass powder acts as a filler, refining the mortar microstructure and further contributing to its overall strength by the filler effects^20,21^.

Kunther, Ferreiro^22^ conducted research on how the Ca/Si ratio affects the compressive strength of cementitious calcium-silicate-hydrate binders. They found that the compressive strength of the samples is influenced by the Ca/Si ratio in the binder, with lower ratios resulting in higher compressive strength due to an increase in the quantity of C–S–H phases. The study also involved the use of MIBA to replace sand in the mortar samples, but the presence of small particles in MIBA can interfere with the Ca/Si ratio between cement and glass powder. The chemical composition of MIBA, which is primarily CaO, can increase the Ca/Si ratio and decrease the compressive strength of the mortar sample.

The morphology of MIBA had an impact on the compressive strength of mortar samples. Due to its porous microstructure and irregular-shaped particles, MIBA has lower density and high absorption properties, which leads to poor bonding properties in the mortar samples. Furthermore, the level of MIBA substitution in the mortar samples played a role in determining the compressive strength. As the level of MIBA substitution increased, there was a decrease in both the compressive strength and density^23^.

This study utilized municipal incinerated bottom ash (MIBA), similar to a study conducted by Lynn, Dhir Obe^3^ which explored the use of MIBA as an aggregate in concrete applications^24^. The findings of the study revealed that substituting fine aggregate with MIBA resulted in lower strength and density in the concrete when compared to natural aggregate. The high porosity and absorption properties of MIBA led to a reduction in the mechanical properties of the concrete mix. The chemical compositions of MIBA, including sulfates, chloride, metallic aluminum, and organics, also impacted the properties of the concrete. The researchers recommended pretreating the MIBA to mitigate the negative effects of these problematic chemical compositions.

Aliabdo, Abd Elmoaty^25^ discovered that incorporating up to 10% glass powder as a cement substitute in concrete could increase the compressive strength of mortar. This was due to the ability of the glass powder to refine the porosity of cement pastes, enhancing the properties of the mortar and concrete. They further explained that the improvement in compressive strength in concrete was a result of the filling, pozzolanic, and hydraulic effects of the glass powder. In addition, Ibrahim^26^ have studied the mixing technique to be use for glass powder for cement substitution, it was found that by mixing the glass powder directly with the cement and aggregate was resulted in higher compressive strength, and density compared with the technique by mixing glass powder with the water for 5 h before mixing with cement and aggregate.This can be concluded that glass powder can be used in cement and concrete applications but the level of glass powder for cement substitution need to be considered since it can effects the compressive strength, tensile strength, and density of the end products^27–29^

The physical and chemical properties of glass powder (GP) and municipal incinerated bottom ash (MIBA) were studied to determine their effects on the physical and mechanical properties of mortar samples. Results showed that substituting cement with GP led to lower compressive strength in the early stages of curing, but it improved the microstructure of the mortar samples at 90 days of curing. This is due to the high silica content of GP, which acted as a filler and refined the microstructure of the mortar samples. On the other hand, substituting sand with MIBA resulted in significantly decreased compressive strength of the mortar samples due to its low specific gravity and poor bonding with the aggregate. High CaO content in MIBA also increased the Ca/Si ratio in the mortar samples, resulting in decreased compressive strength. However, water absorption in mortar samples was lower when MIBA was used to substitute sand at 10 and 20%. The optimal ratio for using MIBA in mortar samples was found to be 20%. Combining GP and MIBA showed that GP20BA10 had the lowest water absorption and the best performance in reducing the porosity of the mortar sample. Increasing MIBA in the mortar sample caused a reduction in CSH and poor bonding in the microstructure, leading to a loss in compressive strength.

## Conclusion

The focus of this research was to find ways to utilize waste materials on Si Chang Island, specifically municipal incinerated bottom ash and glass waste, which are not adequately managed due to waste management limitations on the island. The author aimed to utilize these wastes by substituting them for raw materials in the production of interlocking paving blocks. The study produced mortar samples to identify the optimal ratios for the interlocking paving block production. The research findings indicate that the physical and chemical properties of municipal incinerated bottom ash and glass powder are suitable for partially substituting sand and cement in the interlocking paving block production. The leaching heavy metals of MIBA were found to be below the TCLP threshold limit, making it a non-hazardous waste that can be further utilized. The mechanical performance of mortar samples varied significantly between those cured for 28 days and 90 days. Several ratios of mortar samples met the compressive strength requirements of the standard for interlocking concrete paving blocks (TIS827-2531) of 40 MPa. The utilization of glass powder and municipal incinerated bottom ash in interlocking paving block production is feasible and environmentally friendly. Although the use of waste materials can decrease the physical performance of the blocks, the partial substitution can mitigate this decrease and make it more practical for interlocking paving block production and other construction applications. The best ratio of interlocking paving blocks in terms of mechanical performance was found to be glass powder 10% for cement substitution and municipal incinerated bottom ash 10% for sand substitution. Overall, this research offers a potential alternative for waste management and value addition to waste materials on Si Chang Island.

## Data Availability

The datasets used and/or analyzed during the current study available from the corresponding author on reasonable request.
